# Development and evaluation of bioadhesive buccal films based on sodium alginate for allergy therapy

**DOI:** 10.1016/j.heliyon.2022.e10364

**Published:** 2022-08-23

**Authors:** Krisztián Pamlényi, Katalin Kristó, Tamás Sovány, Géza Regdon jr.

**Affiliations:** University of Szeged, Institute of Pharmaceutical Technology and Regulatory Affairs, Eötvös u. 6., H-6720, Szeged, Hungary

**Keywords:** Innovative drug delivery system, Buccal polymer film, Sodium alginate, Antihistamine, Allergy

## Abstract

Buccal drug administration is a less explored area, therefore researchers and companies focus on its research because of its innovative potential and opportunities. Buccal polymer films (patches) are considered to be an innovative form and have a great number of advantageous properties. Firstly, patients who suffer from swallowing problems and children can also apply them. The active pharmaceutical ingredient enters the systemic circulation directly without degradation and transformation. The aim of this study was to formulate buccal films with sodium alginate (SA) because it is a rarely used, innovative polymer for the formulation of buccal films. The mechanical, chemical properties and dosage forms of the prepared films were investigated with different methods. To formulate the films, cetirizine dihydrochloride (CTZ) was used as model drug, and glycerol (GLY) was added to make the films more elastic. The samples were prepared and stored at room temperature. As a result, it can be seen that the mechanical properties of all film compositions show good results, especially breaking hardness. The films with high SA concentration containing CTZ had appropriate mucoadhesion forces, so these samples are suitable for application on the buccal mucosa. The results of dissolution confirmed this finding. Finally, it can be said we formulated fast dissolving films and it can be concluded that the films prepared with 3% SA concentration containing 1% and 3% GLY can be recommended for buccal application.

## 1 Introduction

Bioadhesive drug delivery systems are becoming increasingly frequent on the market. One such system is buccal tablets, films and gels. This administration route is easy to use, so patient compliance is better than in the case of the intravenous or per os route, etc. [1]. Children, elderly people and people who suffer from swallowing problems can also use it [2]. A further advantage of this alternative route is that the active pharmaceutical ingredient can enter the systemic circulation without transformation because it avoids the first-pass effect of the liver [3]. Furthermore, local and fast systemic effects can be achieved with these systems, therefore they can be used in emergencies such as hypertensive crisis or anaphylactic shock, and also in chronic illnesses like asthma, allergy, hypertension or other chronic diseases [4, 5, 6, 7].

Mucoadhesion is the process when the materials and the mucin of the mucous membrane are held together for a long time by attractive bonding. It is defined as bioadhesion when both connecting materials are biological. Mucin is a hydrophilic macromolecule and possesses a great number of hydrogen atoms and hydroxyl groups and can therefore create primary (covalent, ionic glycosides, ester bonding) and secondary (hydrogen bonding, van der Waals forces) bonding with biological structures [8, 9]. Mucoadhesion includes several types of bonding, the major categories are adsorption theory, diffusion theory, wetting theory, fracture theory, and electrostatic theory [10]. Mucoadhesion force and the time of mucoadhesion are also important for the application because if one of these two parameters is not sufficient, the absorption of the active pharmaceutical ingredient (API) cannot occur.

The first step in the development is the selection of the polymer material with mucoadhesive properties. Many polymers are available with different parameters [11]. Polymers can be from natural sources, semi-synthetic, and synthetic. The most commonly used polymers are cellulose derivatives such as hydroxypropyl cellulose (HPC), hydroxypropyl-methylcellulose (HPMC), but chitosan, sodium alginate (SA), polyacrylic acid (PAA) and polyethylene glycol (PEG) are also common [12, 13, 14, 15, 16]. Plasticizers are also important additives in the formulation. They can increase the elasticity of the films and make the application easier, such are, for example, glycerol and mannitol [17]. In addition, many additives can be applied in polymer films, such as solvents, taste-masking agents, permeation enhancers, saliva-stimulating agents, etc. [18, 19, 20].

In the literature, there are some studies that investigated the effect of the polymer material and the plasticizer or any other ingredient in films, but in these studies the authors did not apply an API despite the fact that the API can remarkably change the properties of films and in terms of use, it would be important to investigate them together [14, 21, 22, 23]. The selected API has to meet some criteria. The applied amount of the API should not exceed 40–50 mg per one dose, it should be soluble in water or in other solvent, and without these criteria formulation is not possible [23, 24].

SA is a rarely used polymer in the formulation of mucoadhesive systems, but it has many advantageous properties [25]. SA is an anionic, natural, non-toxic, biodegradable, biocompatible polymer with appropriate mucoadhesive properties [26, 27]. It can be extracted from brownseeds. SA has many hydroxyl and carboxyl groups, which allow it to create bonding with the mucin of the buccal mucosa [28].

In our previous work, we formulated buccal films with HPMC and SA [27]. HPMC is a conventional and much studied excipient in pharmaceutical technology, therefore we mixed it with SA in that work [27]. In our current project, we used only SA as a polymer film-forming agent without HPMC because the film-forming properties of SA seem to be promising based on our previous work. The aim of the current project was to investigate the film-forming ability of SA and to formulate polymer films from SA which can be applied on the buccal mucosa. This would be a novel additive from the aspect of administration. Our further aim was to incorporate other components in the films, such as glycerol (GLY) and cetirizine dihydrochloride (CTZ). GLY was used as a plasticizer, while CTZ was the model drug. It is a second-generation (non-sedative) antihistamine and a common drug in the oral treatment of allergy [29]. Another purpose of our work was to investigate the mechanical, *in vitro* mucoadhesive and physical-chemical properties of prepared films. In addition, we studied the effect of the plasticizer and the API on the mechanical properties of these films.

## 2 Materials and methods

### 2.1 Materials

SA (Biochemica GmbH, Germany) (10,000–600,000 g/mol) was used as a film-forming agent in the preparation of buccal films. GLY 85% (w/w%) was added to the film as a plasticizer (Ph. Eur. 8.). CTZ (Ph. Eur. 8.) was applied as an active ingredient in the polymer films. It was a gift from Extractum Pharma Pharmaceutical Manufacturing, Marketing and Consulting Inc. Distilled water was used as a solvent.

### 2.2 Preparation of the films

Buccal films were prepared at room temperature with the solvent casting method. At the beginning of preparation, SA (2, 3 w/w%) was dissolved in distilled water and mixed (900 rpm) at room temperature. After solvation, CTZ was added to the polymer solution (0.5523 g/100 g solution) for 8 h. In the third step, GLY (1, 3, 5 w/w%) was incorporated into the solution the following day. Mixing was decreased to 100 rpm for 3 h to make the air bubbles disappear from the solution. The solution was cast on Teflon surface in rubber rings with a diameter of 6.7 cm, with 10 g of solution/ring, then the solvent was evaporated at room temperature (25 ± 0.5 °C) for 72 h. The Teflon surface was selected because the films can be removed easily from the surface and the films do not stick to it. At the same time, Teflon is an inert material, no chemical interactions can take place between the film components and the surface. The dried polymer films were removed from the Teflon surface, were put in closed containers and were stored at room temperature during the investigations. The preparation method was the same for all films to ensure the same conditions.

As can be seen, Table 1 contains Samples 1–18. Four compositions contained 4% SA (Sample 3, Samples 16–18). The preparation of these samples was difficult because of their high viscosity due to the high concentration of the polymer. Also, these samples had several disadvantages in terms of application, such as large thickness and breaking hardness, low flexibility. Therefore no further investigations were done with Sample 3, Samples 16–18.

**Table 1:** Composition of prepared films.

Samples	SA (w/w%)	GLY (w/w%)	CTZ (10 mg)
1	2	-	-
2	3	-	-
3	4	-	-
4	2	1	-
5	2	3	-
6	2	5	-
7	3	1	-
8	3	3	-
9	3	5	-
10	2	1	**+**
11	2	3	**+**
12	2	5	**+**
13	3	1	**+**
14	3	3	**+**
15	3	5	**+**
16	4	1	-
17	4	3	-
18	4	5	-

Table 1 shows the prepared polymer films of different compositions. Samples 10 to 15 are marked in grey because these films contain CTZ.

### 2.3 Thickness of the films

The thickness of the polymer films was measured with a screw micrometre (Mitutoyo Co. Ltd, Japan), sensitivity was 0.001 mm. Six points were selected randomly from all films (n = 6). The means and standard deviations (SD) were evaluated from these data.

### 2.4 Breaking hardness of the films

**Figure 1:**
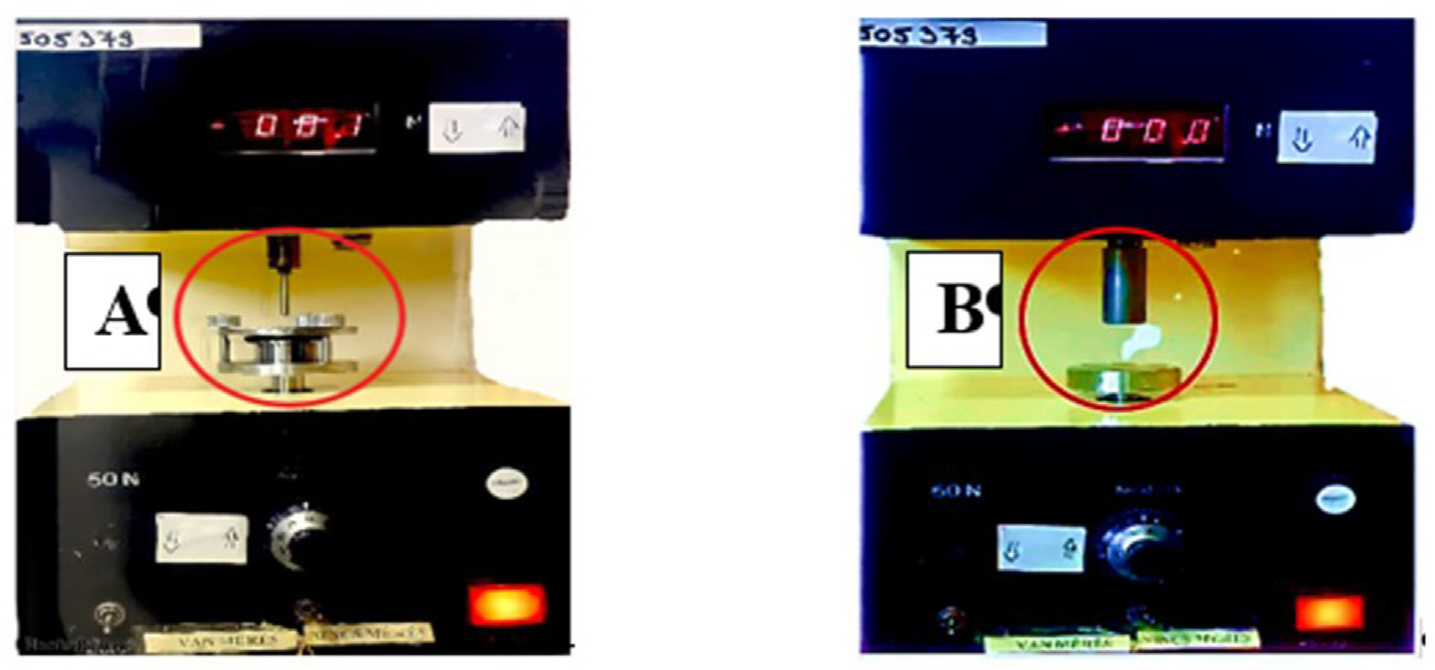
Schematic picture of the self-developed texture analyser. A measures the breaking hardness of films, while B measures the mucoadhesion force of films [31].

Breaking hardness was tested with a self-developed device and software. The device, seen in Figure 1, and the software were developed at our institute [21, 30]. The device has two different types of sample holder as probes (needle-like probe, rod-like probe). The equipment has a fix disc and a vertically moving jowl. Force, force-displacement and time can be registered. A different sample holder can be applied depending on the test. The breaking hardness of the films can be examined with the needle-like probe (its area was 201 mm^2^). At the beginning of the test, the sample was fixed on the bottom part of the equipment and the probe was lowered at constant speed (20 mm/min). The probe moved towards the film and finally it broke the film. The equipment detected the time, force and deformation during the test. The test was finished when the film was broken. The measuring range was 0–200 N, the sampling rate was 50 Hz, the output was 0–5 V, and the sensitivity was ±0.1 N [14]. The test was repeated six times (n = 6) for each film. Tablets, pellets, films can also be investigated with this equipment. The means and standard deviations were calculated.

### 2.5 In vitro mucoadhesivity study

Mucoadhesion was tested with the same self-developed analyser with different parameters and modifications. During the investigation, the rod-like probe was used as sample holder with a diameter of 5 mm. A double-sided adhesive tape was put on the surface of the sample holder, and the polymer samples were fixed on the other side of the adhesive tape. A 35-mm-diameter disc was fixed on the bottom part of the equipment. To this area, 40 μl of freshly prepared mucin dispersion was injected from measurement to measurement. The mucin dispersion was prepared in situ before the investigation, 1 g of mucin was mixed in 10 g of distilled water (10 w/w%). The rod-like sample holder was moved downward and squeezed to the bottom disc, which contained the mucin, with 30 ± 0.1N for 30 s. This constant state part, which can be seen in the force-time curve, is the first phase of the study. During the second phase, the sample holder went upwards, and the force was decreased until the sample started to separate from mucin, which can be detected as a well-defined peak in the force -time curve. The peak maximum indicates mucoadhesion force. The test was repeated five times (n = 5) and the means and standard deviations were calculated.

### 2.6 HATR FT-IR spectroscopy measurement

The FTIR spectra of the raw materials and the polymer films were investigated with an Avatar 330 FT-IR apparatus (Thermo-Scientific, USA). The equipment was coupled with a Zn/Se HATR (horizontal attenuated total reflectance) plate. The films were put directly on a clean crystal of the apparatus. The applied spectral range was 600–4000 cm^−1^ during the investigation. The spectra were collected from 128 scans to obtain smooth spectra, at the spectral resolution of 4 cm^−1^ with CO_2_ and H_2_O correction. The aim of the FTIR measurement was to investigate and determine the chemical interactions between the components of the films. FTIR spectroscopy demonstrated the different interactions that take place in the polymer film system, as can be seen in the results. The explanation of physical phenomena usually derives from chemical processes, therefore it is required to know the chemical background. We tried to find a connection between the chemical interactions and the other properties of the films.

### 2.7 Thermoanalytical measurement (thermogravimetric analysis (TGA), differential scanning calorimetry (DSC))

The thermoanalytical measurement of the prepared films was carried out with a Mettler-Toledo TGA/DSC1 instrument (Mettler Toledo, Switzerland). Small pieces of films (approximately 10 mg) were placed in aluminum pans (100 μL), and were inserted into the instrument. During the measurement, the start temperature was 25 °C and the end temperature was 500 °C. The heating rate was 10 °C/min. The samples were investigated in flowing nitrogen atmosphere, the flow rate was 50 ml/min. The curves were evaluated from the average of two parallel measurements with STARe software [32, 33].

### 2.8 Dissolution test

Pieces of film of the size of 2 × 2 cm (containing 10 mg of cetirizine dihydrochloride) were used in the dissolution test. Erweka DT700 dissolution equipment with basket tester was used in the investigation. The mixing speed was 100 rpm and the temperature was 37 °C. The dissolution medium was 900 ml of phosphate buffer (pH = 6.8). Aliquots were 5 ml and were analysed in 5, 10, 15, 20, 30, 40, 50, 60, 90, 120 min with Genesys 10S UV-VIS (Thermo Fisher Scientific, USA) UV-spectrophotometry at λ = 207 nm, which was a sharp and characteristic peak of the raw material CTZ. This peak was determined to create a calibration solution from CTZ with distilled water. The UV spectra of the raw materials were collected and the peak of the raw materials did not interfere with the peak of CTZ at 207 nm.

### 2.9 Statistical analyses

The significance test of breaking hardness and *in vitro* mucoadhesivity was evaluated with Microsoft Excel (version 15, Redmond, Washington USA) as software. A Two-Sample T-Test was applied. The test was run six times for each sample. In all cases, the samples were compared to the composition without CTZ. In each case, we used a significance level p < 0.05. Significance is labelled as ns = p < 0.05; ∗

## 3 Results

### 3.1 Thickness of the films

Table 2 shows the thickness of the films prepared in different compositions. As we can see, the thinnest film is Sample 1 (67.45 μm), which was formulated without GLY and CTZ, and it contained the least amount of SA. The concentration of SA can increase thickness, and a similar effect can be observed in the case of raising the GLY concentration. Sample 3 is thicker (212.43 μm) than Sample 2 (99.34 μm), and Sample 9 (177.38 μm) is also thicker than Sample 7 (106.63 μm). CTZ can also increase thickness, the films containing CTZ have greater thickness than their CTZ-free counterparts (Sample 10–105.23 μm, Sample 4–98.29 μm). However, it can be stated that GLY increased thickness to a greater extent than CTZ due to the fact that GLY has a water retention effect [34]. At the same time, SA can also increase the thickness of the films because SA and CTZ can enhance the dry matter content of the films.

**Table 2:** Thickness of the polymer films.

Samples	Thickness (μm)	Standard deviation of thickness (μm)
**1**	67.45	±8.53
**2**	99.34	±10.38
**3**	212.43	±4.92
**4**	98.29	±6.38
**5**	135.66	±9.86
**6**	199.33	±7.53
**7**	106.63	±8.42
**8**	123.12	±3.36
**9**	177.38	±6.92
**10**	105.23	±5.18
**11**	148.03	±13.88
**12**	212.32	±3.43
**13**	158.05	±4.34
**14**	194.14	±2.97
**15**	253.17	±11.30

### 3.2 Breaking hardness of the films

**Figure 2:**
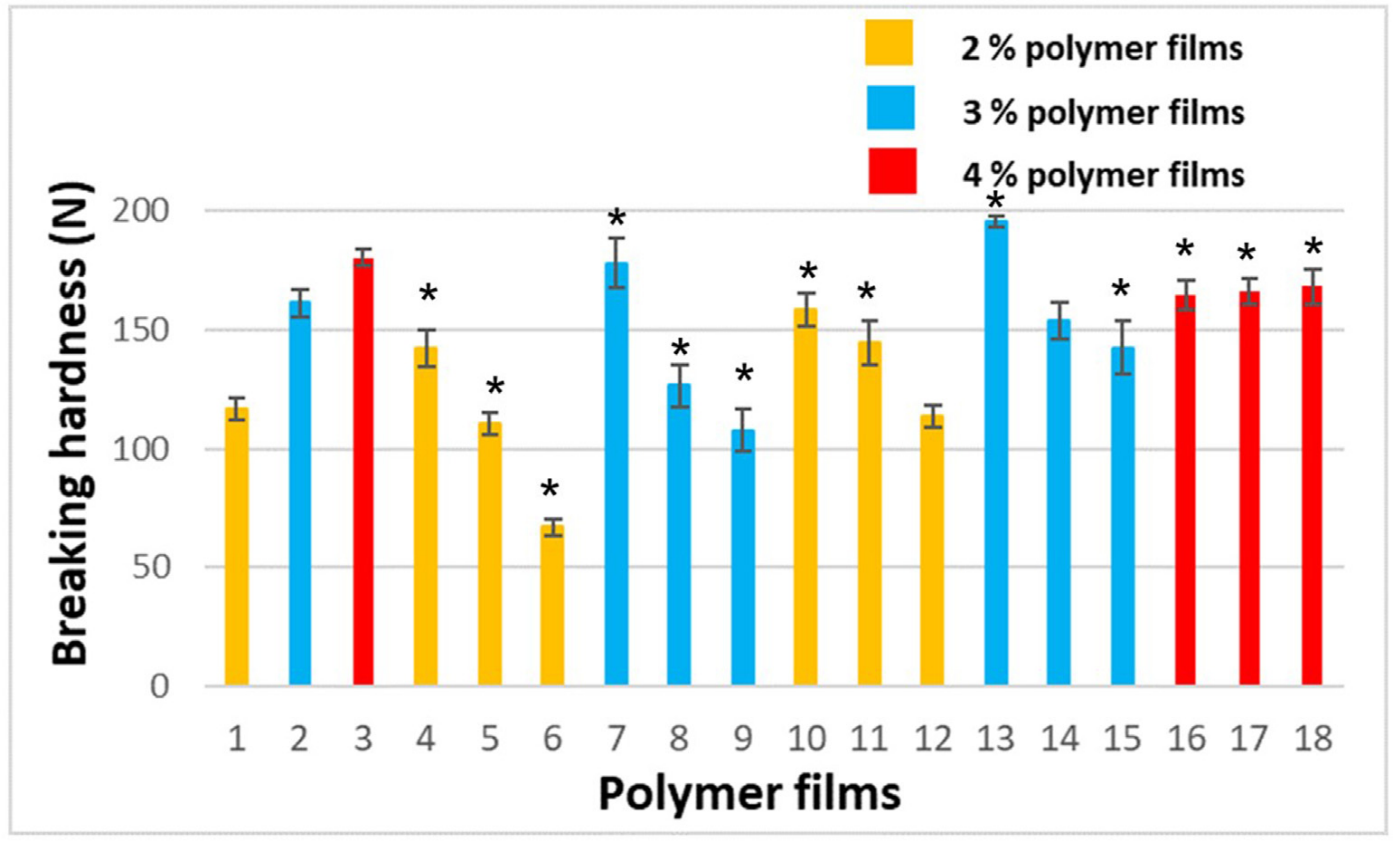
Breaking hardness of the prepared films.

The breaking hardness of films is an important investigation with respect to application. The patient has to place the film on the buccal mucosa. During application, the patient exerts force on the film, therefore the film must have sufficient breaking hardness to resist the force and have adequate flexibility. During the preformulation study, we tried to simulate the application force to the mucosa with our fingers. The moving part of our equipment was removed and the film was pressed with fingers, with a force similar to that applied by the patients when pressing it to the mucosa. We concluded that a force of 120 N is sufficient to create mucoadhesion to the buccal mucosa. So films must have breaking hardness greater than 120 N to be appropriate for application. Figure 2 shows the breaking hardness of films of different compositions. The significance results are marked with ∗ (∗; p < 0.05). The breaking hardness of SA-based films is high because the chains of SA can create a strong cohesive structure [27]. This hardness was decreased by GLY, probably GLY increases the bonding distance between the molecules, therefore the film has a lower breaking point despite the higher amount of plasticizer. Before the breaking point, these films were more elastic due to the plasticizer. CTZ can also influence this parameter of films, it can enhance the strength of films due to creating bindings with SA and GLY. The investigation of Samples 16–18 reveals that in the films containing 4% polymer concentration the effect of GLY cannot prevail, presumably the large number of polymer chains can create a very strong structure and GLY cannot degrade the structure. During preparation, this great hardness was also experienced because of high viscosity. Overall, it can be said that most of the compositions have appropriate breaking hardness because all the films containing CTZ have greater breaking hardness than 120 N, except for Sample 12.

### 3.3 In vitro mucoadhesivity study

**Figure 3:**
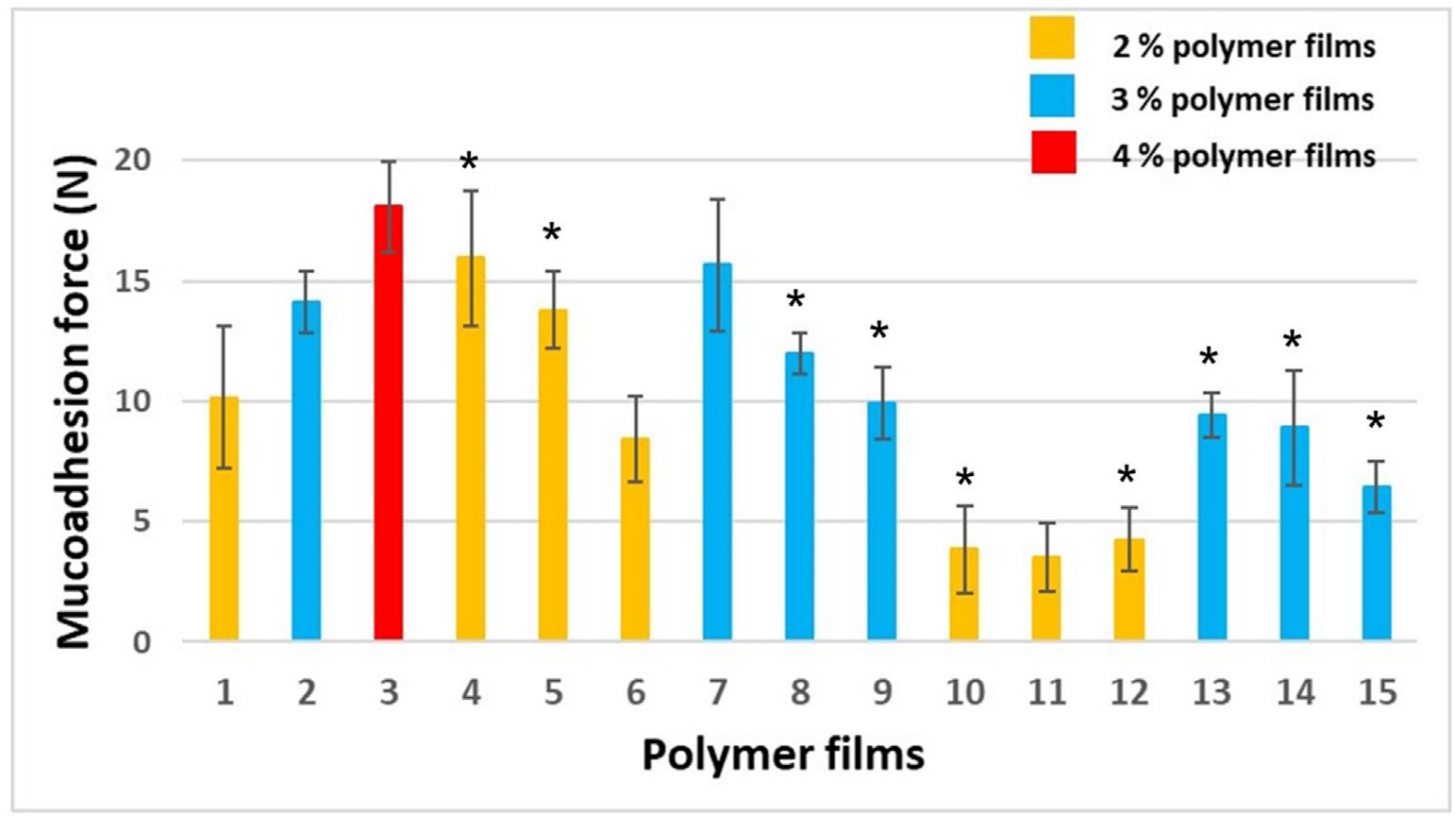
Mucoadhesion force of the prepared films.

*In vitro* mucoadhesivity study is another important investigation in terms of application because the films must adhere to the mucosa for the absorption of the API. Figure 3 shows the results of this study. The significance results are marked with ∗ (∗; p < 0.05).

As SA is a mucoadhesive polymer, it can noticeably increase the mucoadhesion force of films due to the high number of free chains which can bind to the mucin of the buccal mucosa. Sample 3 has the largest mucoadhesion force from among the films of different compositions. The 2 and 3% films, which contain GLY but no CTZ, have similar mucoadhesive properties. The addition of a low amount of GLY to the film can cause higher mucoadhesion force. This observation can be explained by the plasticizer effect of GLY. As a result, the films become more elastic and fit better with the probe and with the bucca than without plasticizer. The higher GLY concentration causes less mucoadhesion force, so GLY reduces the mucoadhesivity of the prepared films. The reason for this conclusion may be that an interaction may occur between the carboxyl group of GLY and CTZ and SA, leaving fewer free carboxyl groups which can bind to the oligosaccharide units of mucin. This observation can be confirmed by the results of FT-IR measurements.

From the mucoadhesivity data, we found that the films with 2% SA concentration have 7.44 ± 0.23 N. The films with 3% SA concentration have a value close to 18 N with our equipment. It is known from the literature that the SA has smaller mucoadhesivity (with our equipment more than 7 N) than cellulose derivatives, but its mucoadhesivity is also sufficient to bind to the buccal mucosa [35]. We drew the limit at 7 N. So if the mucoadhesivity of the different compositions is larger than 7 N, it means they have sufficient force for buccal mucoadhesion.

Films containing CTZ have less mucoadhesion force compared to their counterparts without CTZ, as can be seen in Figure 3. It can be concluded that CTZ decreases the mucoadhesion force of films. The 2% polymer films containing CTZ have very low mucoadhesivity, their force is less than 5 N. Because of the low forces, these compositions are not suitable for application on the buccal mucosa. The 3% polymer films containing CTZ have better mucoadhesivity properties, their force is almost 10 N, except for Sample 15 containing 5% GLY.

### 3.4 FT-IR spectroscopy measurement

**Figure 4:**
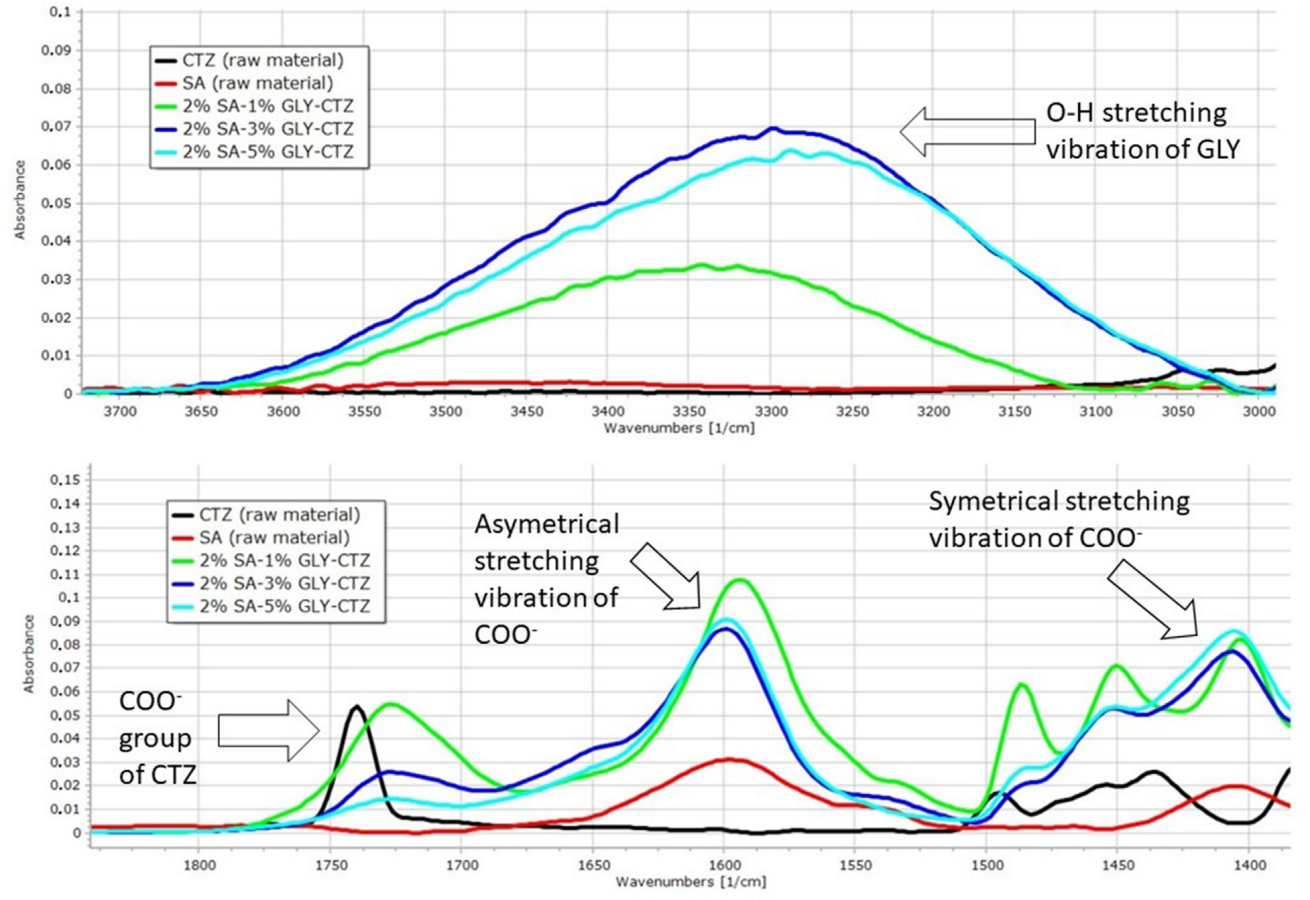
Spectra of raw materials and 2% polymer films.

**Figure 5:**
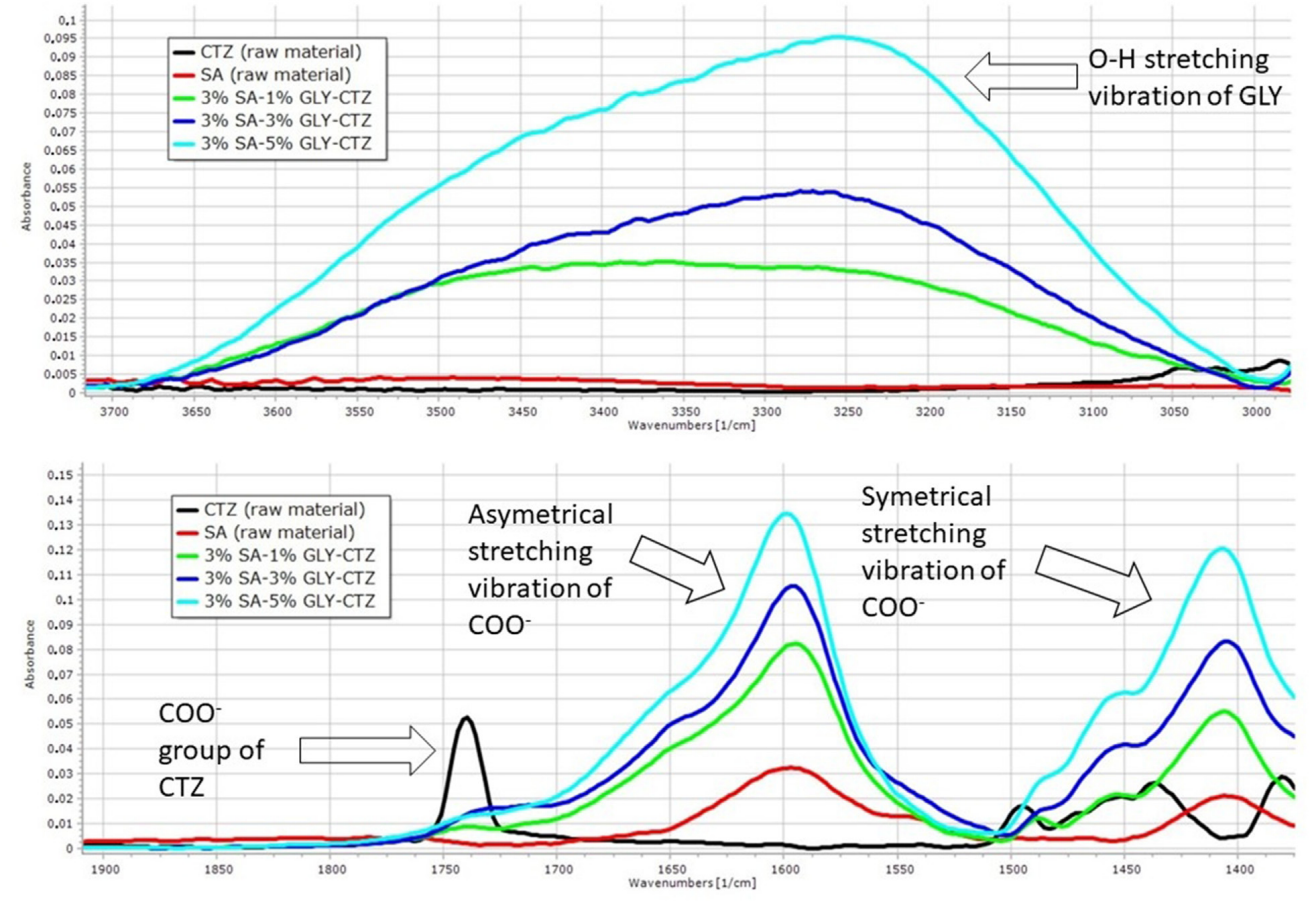
Spectra of raw materials and 3% polymer films.

FT-IR spectroscopy as an interaction study was carried out to obtain information about the interactions between the API and other excipients in the film. The results of the FT-IR investigation can be seen in Figure 4 and Figure 5. In both spectra, there is a peak at 3340 cm^−1^, which corresponds to the O–H stretching vibration of GLY [36]. This peak shifts to the lower frequency zone and its intensity increases with higher GLY concentration. This observation can be attributed to weak interactions, intermolecular hydrogen bonds between SA, GLY and CTZ.

The peak at 1739 cm^−1^ may appear, which determines the carboxyl group of the API [37]. This peak disappears as the amount of the polymer and the plasticizer increases. Figure 4 shows a peak shifted to the lower wavenumber in the case of the 2% polymer films, and the shifted peak is larger when increasing the GLY concentration. With 3% SA and high concentrations of GLY, this peak totally disappears and becomes unidentifiable (Figure 5). This phenomenon shows a strong interaction (hydrogen bonding) between the carboxylic group of CTZ and the OH^−^ group of the polymer and the plasticizer.

In the spectra, there are two characteristic peaks at 1586 cm^−1^ and 1412 cm^−1^. These peaks represent the asymmetrical and symmetrical stretching vibration of COO^−^ groups. As a function of GLY concentration, the intensity of the peak may increase in the case of films with 3% polymer concentration, as shown in Figure 4 and Figure 5. This phenomenon is due to the interaction between CTZ, SA and GLY, as can be seen at 1739 cm^−1^ also. The reason for this observation is that the 3% polymer films contain more free COO^−^ groups than the 2 % polymer films, therefore a higher peak can be detected in this area in Figure 5.

The results of the FT-IR measurement can be related to the mechanical properties of the films and the dissolution test. As can be seen from the FT-IR measurements, at 1739 cm^−1^ a strong interaction can occur in the 3% polymer films, which means these films have a cohesive, stable structure with higher breaking hardness and higher mucoadhesivity than the 2% polymer films. At the same time, these strong interactions can slow drug release because the hydrogen bond can cause a stable structure and it is more resistant to the degradation effect during the dissolution test. The effect of GLY concentration on drug release is connected to the interaction at 3300 cm^−1^, so the films with the highest GLY concentration can form the strongest interactions, therefore the dissolution is the slowest in the case of this film composition.

As a conclusion, we focused on identifying the chemical interactions between the components of films with FT-IR spectroscopy. It is important to know because chemical interactions can influence the physical parameters of the film. From our results, it can be concluded that strong interactions can occur between SA, GLY and CTZ. Typically, hydrogen bonds can be formed in the films, and the strength of the bond depends on the polymer concentration. These interactions affect mostly the dissolution of the API. The purpose of the FT-IR measurement was achieved.

### 3.5 Thermoanalytical measurement (TGA, DSC)

**Figure 6:**
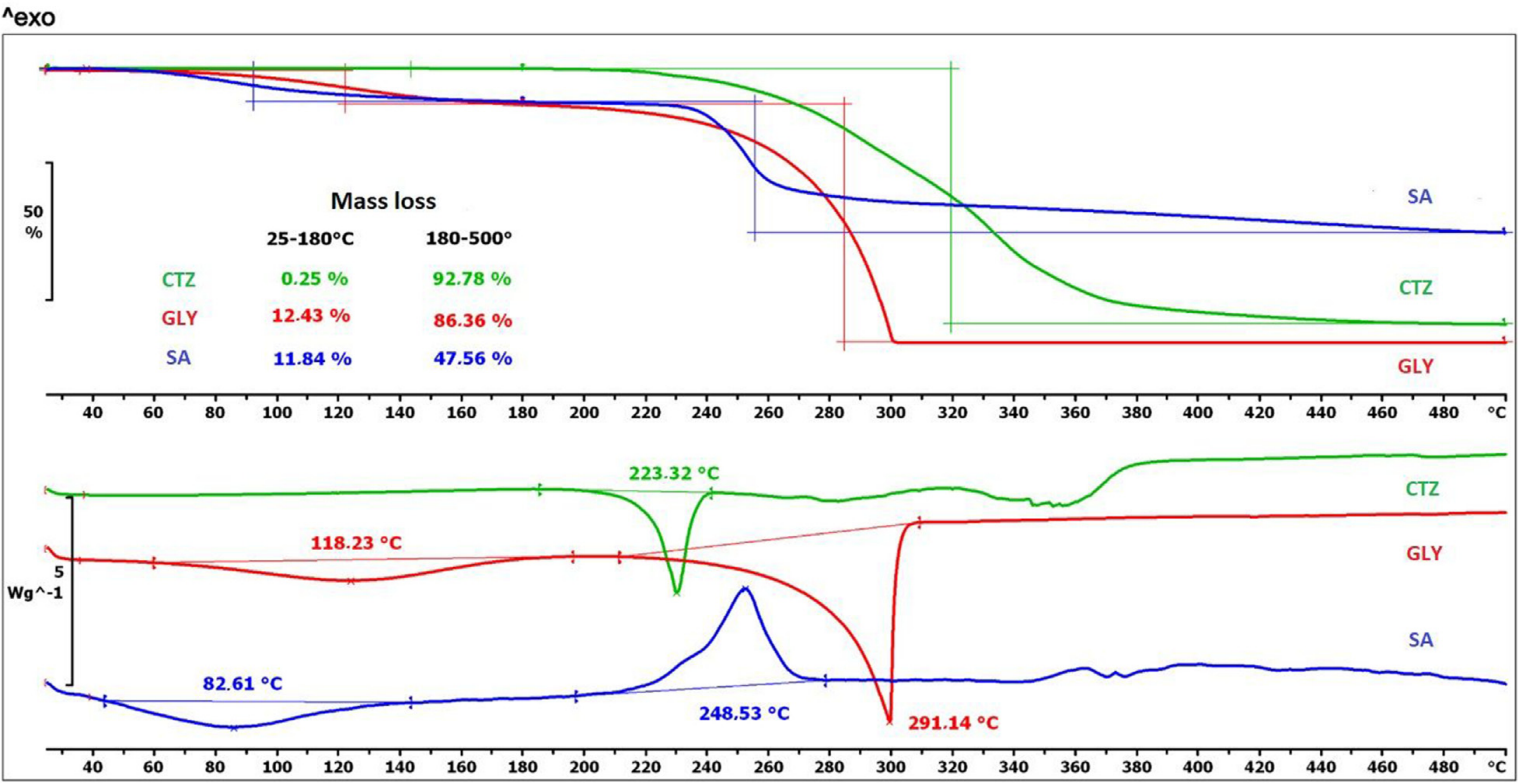
Thermal properties of raw materials as shown by TGA and DSC curves.

In Figure 6 the TGA and DSC curves of the raw materials can be seen. The TGA curves in Figure 6 show that the mass loss of the film-forming agent is 11.84% until 180 °C, and 47.56% until 500 °C. The decomposition process starts from 75 °C. For GLY, the mass loss is almost similar until 180 °C, but it is more than 86% until 500 °C. The mass loss curve of CTZ reveals that the decomposition starts above 200 °C, so CTZ can be said to be a thermostable API.

The DSC curve of SA shows an endothermic peak from 40 °C to 150 °C and an exothermic peak is visible from 200 °C to 280 °C. For GLY, 2 peaks can also be detected. The first peak appears at 118.23 °C, it is an endothermic peak. The second peak starts from 215 °C and ends at 320 °C. This peak indicates the decomposition of GLY. The decomposition of CTZ starts after the melting point, which can be seen at 223.32 °C, followed by the general decomposition of CTZ.

**Figure 7:**
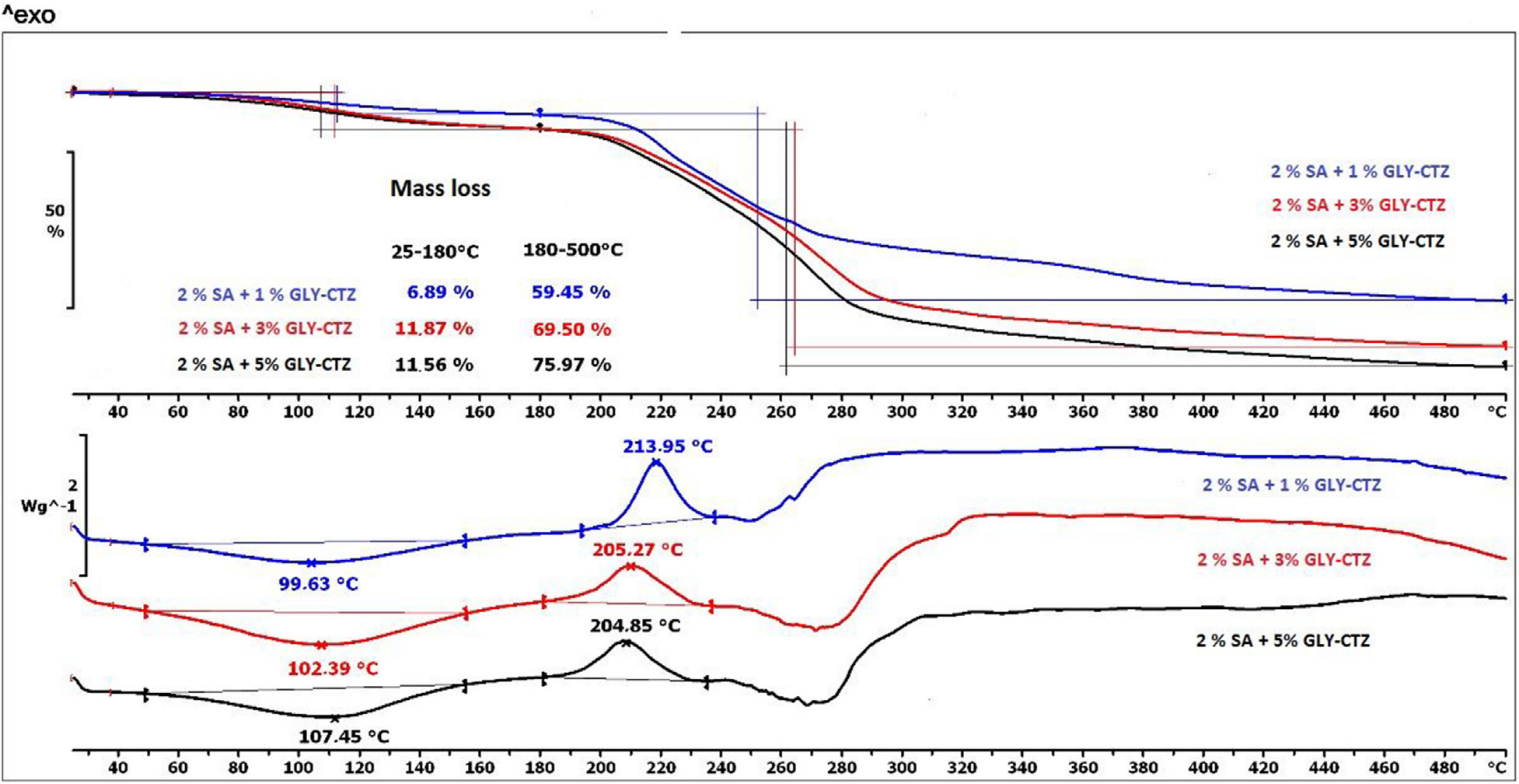
Thermal properties of the 2% polymer films as shown by TG and DSC curves.

**Figure 8:**
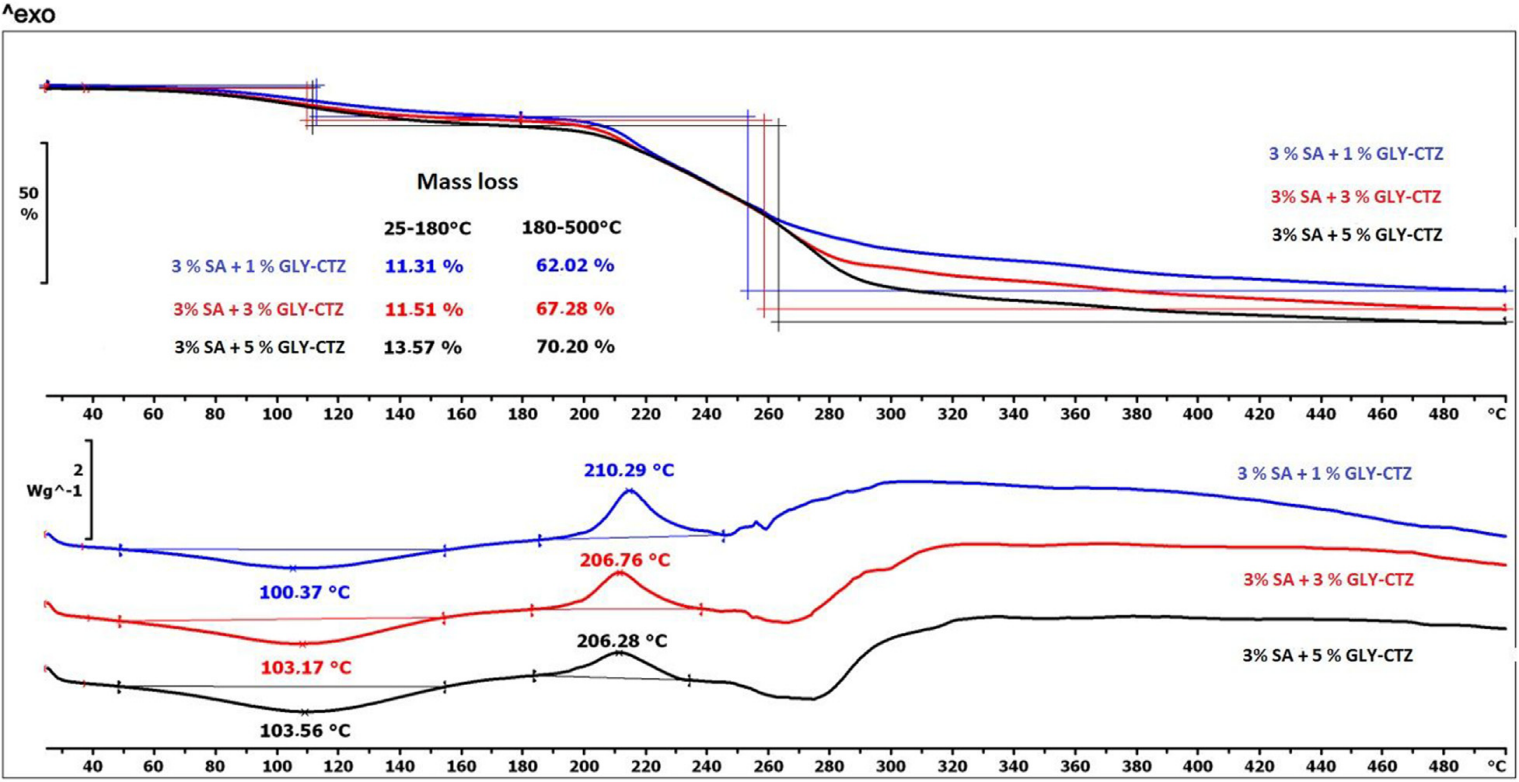
Thermal properties of the 3% polymer films as shown by TG and DSC curves.

After the examination of the excipients, we wished to investigate the behaviour of the prepared films and explore the interaction between the excipients. The results of the thermal behaviour of films can be found in Figure 7 and Figure 8. Figure 7 shows the thermal behaviour of 2% polymer films, and in Figure 8 the thermoanalytical curves of 3% polymer films can be seen. The decomposition of buccal films can take place in two steps. In the first step (until 180 °C), it is visible that films with the lowest GLY concentration (Sample 10) have the lowest mass loss, which can be explained by the lower water content of films with low GLY concentration. In the case of larger GLY and SA concentrations, which result in a higher water content, there is no significant difference in the mass loss of Samples 11–15.

In the second step of Figure 7 and Figure 8, the same observations can be made, but the values of the mass loss of the films with different compositions are higher and can be detected from 60% to 75% due to the remarkable decomposition of CTZ and GLY and a little bit of SA.

Two characteristic peaks of SA can be found in the DSC curves of the films with different compositions (Figure 7 and Figure 8). The first peak shifts towards higher temperature with increasing GLY concentration. This observation may reveal a moderate interaction between the excipients of the films, which may indicate the presence of hydrogen bonds. This fact confirms the results of FT-IR spectroscopy. The second (exothermic) peak of the DSC curves also shows a shift with increasing GLY concentration, the exothermic peak moves towards the lower temperature. This correlation appears to be notable for the films with 1% and 3% GLY concentrations, but it is not noticeable for films with medium and high GLY concentrations. The correlation can be applied to films with 2% and 3% SA concentrations.

In summary, the decomposition processes generally start from 70 °C, so the polymer films can be considered thermally stable up to this temperature.

### 3.6 Dissolution test

**Figure 9:**
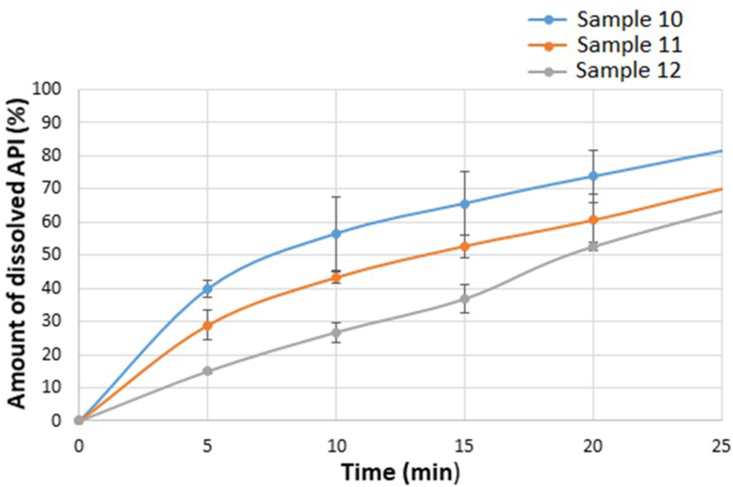
Dissolution curves of the 2% polymer concentration films under 20 min.

The dissolution study is one of the most important dosage form tests. The results of the dissolution study of the films with 2 % polymer concentration are shown in Figure 9. During the whole test (120 min), the total amount of CTZ dissolved from the different compositions of the films. In the first 20 min, the largest amount dissolved from the composition containing less GLY, around 73% more than from films with a higher GLY content. Less API could dissolve from the film with 5% GLY concentration, in this case less than 55%. GLY can remarkably reduce the speed of dissolution. This fact is the most likely related to the stronger bonding between the components.

**Figure 10:**
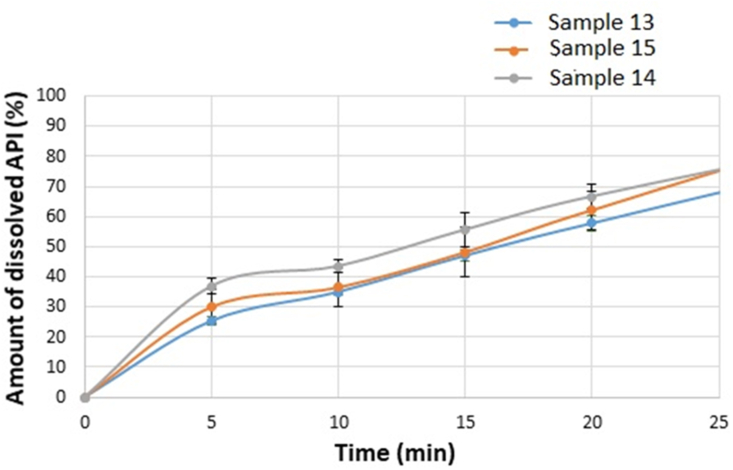
Dissolution curves of the 3% polymer concentration films under 20 min.

Figure 10 shows the dissolution of the 3% polymer films. From these films, the total amount of CTZ could also dissolve during the whole test. There is no significant difference between the amount of the dissolved API and the different plasticizer concentrations of the 3% polymer films. A difference can be detected in the shape of the curves. In the case of 2% polymer films, the curves are comparable to a saturation curve without steady state stage. The curves of the films with 3% polymer concentration have a step between 7 and 10 min, in this period steady state can be detected, which can also be explained by the structural relationships between SA, GLY and CTZ, which is supported by FT-IR spectroscopy measurement. A more stable structure can be created, therefore it takes time for the cohesive and adhesive structure to degrade and for the drug to be released.

## 4 Conclusion

In our present research, we focused on the film-forming ability of SA and on formulating buccal films with SA. In the films, CTZ was applied as a drug. The mechanical properties of the films showed that SA can create a strong, cohesive structure, and due these properties all compositions had a very large breaking hardness. It can be concluded from the result that the amount of SA and CTZ can increase this film parameter, but GLY reduces it.

From the mucoadhesion study, we can observe the good mucoadhesive properties of SA, while GLY and CTZ reduce the mucoadhesion force of films. The films with 2% polymer concentration containing API had no satisfactory mucoadhesivity, so Samples 10–12 cannot be used on the buccal mucosa, whereas the films with 3% polymer concentration containing CTZ had fine mucoadhesivity property.

By the FT-IR measurement, weak and strong interactions between the different excipients can also be detected. These interactions can typically be identified as hydrogen bondings, which confirmed the results of the mechanical properties of the films, especially breaking hardness.

Thermal analysis can also detect interactions between the different materials in the films. This interaction determines that hydrogen bondings can be created in the films. The observation of interaction between the excipients was proved with two different measurements. Due to this phenomenon, the thermal stability of films can increase in the first place, but it can also increase mechanical stability. The films are thermally stable up to 70 °C, so it is possible to increase the temperature of preparation to speed up the production.

Overall, it can be stated that we formulated a fast dissolving buccal film from SA and found promising compositions, especially Sample 13 (3 % SA+1% GLY-CTZ) and Sample 14 (3 % SA+1% GLY-CTZ), which are suitable and possible to apply as a buccal drug delivery system.

## Declarations

### Author contribution statement

Krisztián Pamlényi: Conceived and designed the experiments; Performed the experiments; Analyzed and interpreted the data; Wrote the paper.

Katalin Kristó: Conceived and designed the experiments; Analyzed and interpreted the data; Wrote the paper.

Tamás Sovány: Analyzed and interpreted the data; Wrote the paper.

Géza Regdon jr: Conceived and designed the experiments; Analyzed and interpreted the data; Contributed reagents, materials, analysis tools or data; Wrote the paper.

### Funding statement

This work was supported by The University of Szeged Open Access Fund (FundRef, Grant No. 5651), was also supported by Richter Gedeon Talentum Foundation (Grant No. 010819) and the ÚNKP-21-3 New National Excellence Program of the Ministry for Innovation and Technology from the Source of the National Research, Development and Innovation Fund (Grant No. ÚNKP-21-3).

Krisztián Pamlényi was supported by Richter Gedeon Talentum Foundation (Grant No. 100108190822).

### Data availability statement

Data will be made available on request.

### Declaration of interest's statement

The authors declare no conflict of interest.

### Additional information

No additional information is available for this paper.
